# Automated classification of fat-infiltrated axillary lymph nodes on screening mammograms

**DOI:** 10.1259/bjr.20220835

**Published:** 2023-09-26

**Authors:** Qingyuan Song, Roberta M. diFlorio-Alexander, Ryan T. Sieberg, Dennis Dwan, William Boyce, Kyle Stumetz, Sohum D. Patel, Margaret R. Karagas, Todd A. MacKenzie, Saeed Hassanpour

**Affiliations:** 1 Department of Biomedical Data Science, Geisel School of Medicine, Dartmouth College, Lebanon, New Hampshire, United States; 2 Department of Radiology, Dartmouth-Hitchcock Medical Center, Lebanon, New Hampshire, United States; 3 Department of Radiology, School of Medicine, University of California, San Francisco, California, United States; 4 Department of Internal Medicine, Carney Hospital, Dorchester, Massachusetts, United States; 5 Geisel School of Medicine, Dartmouth College, Lebanon, New Hampshire, United States; 6 Department of Epidemiology, Geisel School of Medicine, Dartmouth College, Lebanon, New Hampshire, United States; 7 Department of Computer Science, Dartmouth College, Hanover, New Hampshire, United States

## Abstract

**Objective:**

Fat-infiltrated axillary lymph nodes (LNs) are unique sites for ectopic fat deposition. Early studies showed a strong correlation between fatty LNs and obesity-related diseases. Confirming this correlation requires large-scale studies, hindered by scarce labeled data. With the long-term goal of developing a rapid and generalizable tool to aid data labeling, we developed an automated deep learning (DL)-based pipeline to classify the status of fatty LNs on screening mammograms.

**Methods:**

Our internal data set included 886 mammograms from a tertiary academic medical institution, with a binary status of the fat-infiltrated LNs based on the size and morphology of the largest visible axillary LN. A two-stage DL model training and fine-tuning pipeline was developed to classify the fat-infiltrated LN status using the internal training and development data set. The model was evaluated on a held-out internal test set and a subset of the Digital Database for Screening Mammography.

**Results:**

Our model achieved 0.97 (95% CI: 0.94–0.99) accuracy and 1.00 (95% CI: 1.00–1.00) area under the receiver operator characteristic curve on 264 internal testing mammograms, and 0.82 (95% CI: 0.77–0.86) accuracy and 0.87 (95% CI: 0.82–0.91) area under the receiver operator characteristic curve on 70 external testing mammograms.

**Conclusion:**

This study confirmed the feasibility of using a DL model for fat-infiltrated LN classification. The model provides a practical tool to identify fatty LNs on mammograms and to allow for future large-scale studies to evaluate the role of fatty LNs as an imaging biomarker of obesity-associated pathologies.

**Advances in knowledge:**

Our study is the first to classify fatty LNs using an automated DL approach.

## Introduction

Obesity, a prevalent global health concern, poses a significant risk factor to an increased morbidity and mortality associated with cardiometabolic diseases, cancers, and other chronic diseases.^1–3^ Traditionally, a body mass index (BMI) >30 kg/m^2^ has been used to assess the risk of obesity-related diseases. However, an increased body of evidence showed that BMI is not a sufficient risk indicator.^4–6^ Instead, ectopic fat deposition—the accumulation of fat within and around organs such as the visceral cavity, liver, and muscle—has emerged as a more accurate predictor of obesity-associated health risks.^4,7,8^

**Figure 1.**
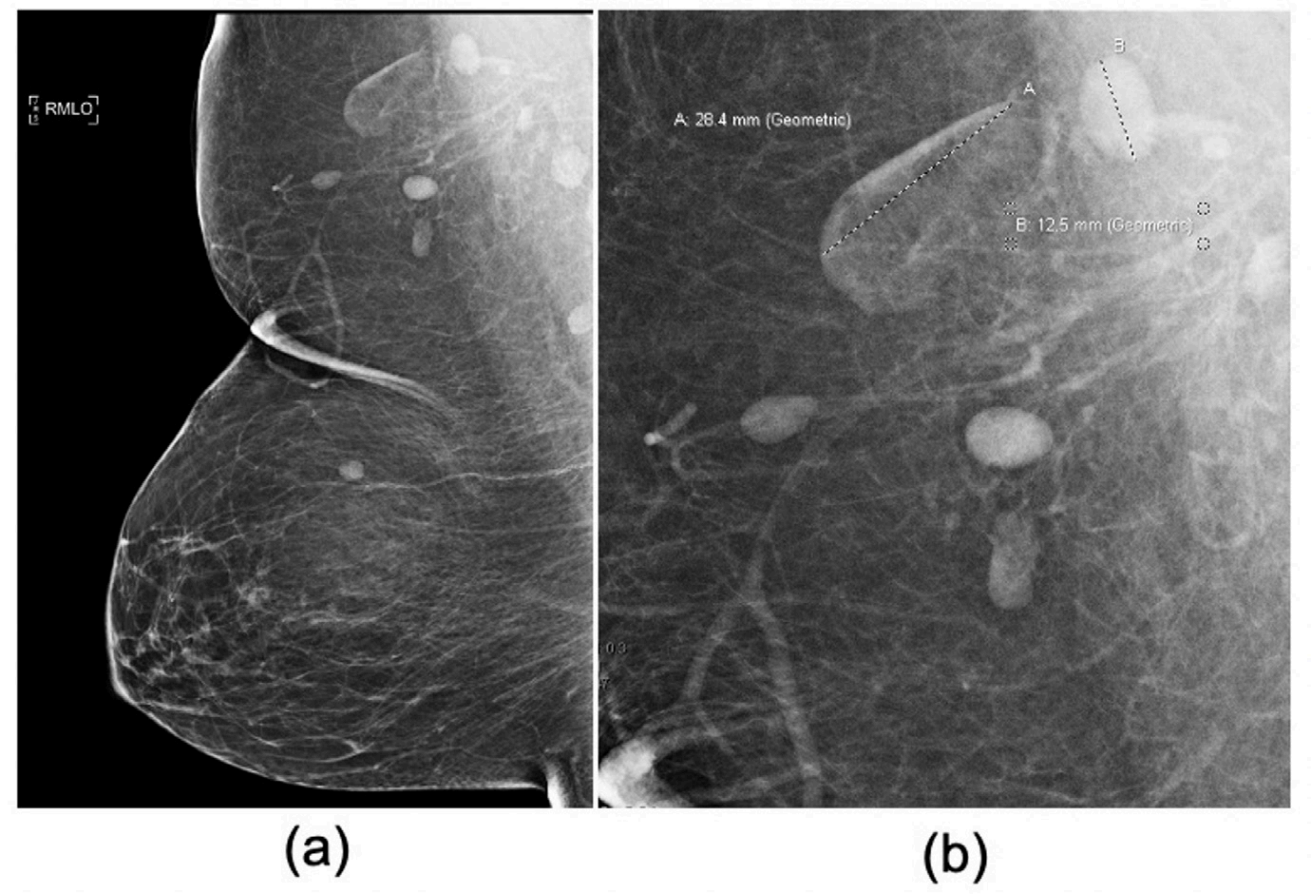
Screening mammogram of a 70-year-old female with benign variable LN morphology. (a) Full-field MLO view of the right breast and (b) magnified view of the right axilla demonstrates two adjacent LNs with differences in central lucency and size and morphology (A: 28.4 mm *vs* B: 12.5 mm) secondary to variable degrees of fat expansion of the LN hilum. We recorded the status as a benign fat-infiltrated LN based on the largest longitudinal axis of the largest visible axillary LN. LN, lymph node; MLO, mediolateral oblique.

Notably, recent studies propose axillary lymph nodes (LNs) as novel ectopic fatty depots, potentially associated with obesity-related diseases.^9,10^ When fat infiltrates the normal LNs, they exhibit variable size and morphology due to the deposition of fat in their hilum. This condition, known as fat-infiltration of LNs, presents as an expanded, lucent hilum that increases the overall size of the LN (Figure 1a).^11,12^ In contrast, normal axillary LNs appear smaller and denser on mammography (Figure 1b).

Fat-infiltrated axillary LNs have gained interest due to their potential clinical implications. Studies have found a correlation between fat-infiltrated axillary LNs and obesity, where obese females demonstrated an increased LN hilum width of 18 mm compared to that of 9 mm among their normal-weight conterparts.^11^ Furthermore, the presence of fat-infiltrated LNs with a size greater than 18 mm has been linked to a higher risk of breast cancer nodal metastasis in obese breast cancer patients, independent of patients’ age, BMI, and tumor characteristics.^9^ Another recent study reported a positive association between fat-infiltrated axillary LNs and the prevalence of Type two diabetes (T2DM) in females with obesity, independent of age and BMI.^10^ Recently, a smaller study reported a decrease in serum lipids in obese females who had a reduction in the size of fatty nodes after bariatric surgery, raising the possibility that fatty LNs are a modifiable risk factor for cardiometabolic disease among people with obesity.^13^ Collectively, these studies underscore the potential of mammographically detected fat-infiltrated axillary LNs as a novel imaging biomarker for advanced breast cancer status and cardiometabolic disease in obese females.

Despite their clinical significance, fat-infiltrated LN assessment in clinical practice remains limited by several pain points. Fat-infiltrated axillary LNs are considered benign normal variants and do not currently necessitate further evaluation, measurement, or analysis during standard clinical mammographic imaging interpretation. The manual measurement and classification of axillary LNs, as currently performed by radiology experts in the previous studies,^9,11,14^ can be time-consuming, laborious, and prone to interobserver inconsistencies.^9,11,13^ The lack of standardization and the substantial human effort required hinder the efficient assessment of fatty LN status on breast images, particularly for large-scale studies aimed at exploring the potential of fat-infiltrated axillary LNs as imaging biomarkers of obesity-associated diseases.

Deep learning (DL) models have shown great promise in addressing such pain points in clinical image analysis. Specifically, convolutional neural networks (CNNs), a class of DL model, have been utilized for medical image analysis and demonstrated exceptional performance in various clinical tasks.^15–17^ CNN approaches can bypass the laborious manual feature extraction steps and directly operate on the input images by learning the indicative patterns for the classification and characterization of the images. Additionally, CNN models that have been pre-trained on vast image data sets and have learned feature extraction on generic images can be subsequently fine-tuned to attain expertise-level performance on medical image data sets that are usually much smaller.^18,19^

In this paper, we aim to investigate the feasibility of employing DL models for fat-infiltrated LN assessments on screening mammograms. Our goal is to develop an automated, end-to-end classifier to streamline the assessment process and facilitate large-scale studies on fat-infiltrated LNs as potential imaging biomarkers for obesity-related diseases. This pioneering effort represents the first attempt to apply DL techniques to automatically evaluate fat-infiltrated LN status on screening mammography, laying the foundation for future research in this area.

## Methods

### Data source for model development

This study, and the usage of human participant data in this project, were approved by the Institutional Review Board (IRB) with a waiver of informed consent. The internal data set used for this study included 886 full-field digital screening mammogram (FFDM) mediolateral oblique (MLO) views with detectable axillary LNs collected through a retrospective review of screening mammograms over an 8-month period at a tertiary care academic medical facility. The detailed data collection process is described in a previous study.^10^ In brief, the largest visible axillary LN on the left or right MLO view was measured and labeled, with a final assessment achieved via a consensus review between two radiology reviewers.^9^ The images were labeled as positive samples if they contained a fat-infiltrated LN larger than 18 mm and with a fat-infiltrated LN morphology, or as negative samples if smaller than or equal to 18 mm. The data set yielded 416 positive samples and 470 negative samples.

### Image pre-processing and internal data set construction

The mammograms were received in the standard Digital Imaging and Communications in Medicine (DICOM) format and came in various orientations and sizes. Images were de-identified by removing the metadata, preserving the pixel values, and fed to a pre-processing workflow implemented in Python (v. 3.8). We applied a contour finding algorithm^20^ to identify the boundary of the breast and masked all areas outside of the boundary. The process removed all text markers and noise outside of the breast region, ensuring a cleaner image for experiments. Through visual inspection of the mammograms, we found that a region of 1500 × 1500 pixels in the axillary region of the mammogram was sufficient to cover all the axillary LNs. Therefore, a 1500 × 1500-pixel patch in the axillary region of each image was extracted and used to construct the initial data set for model development.

### Oversampling of non-axillary patches

**Figure 2.**
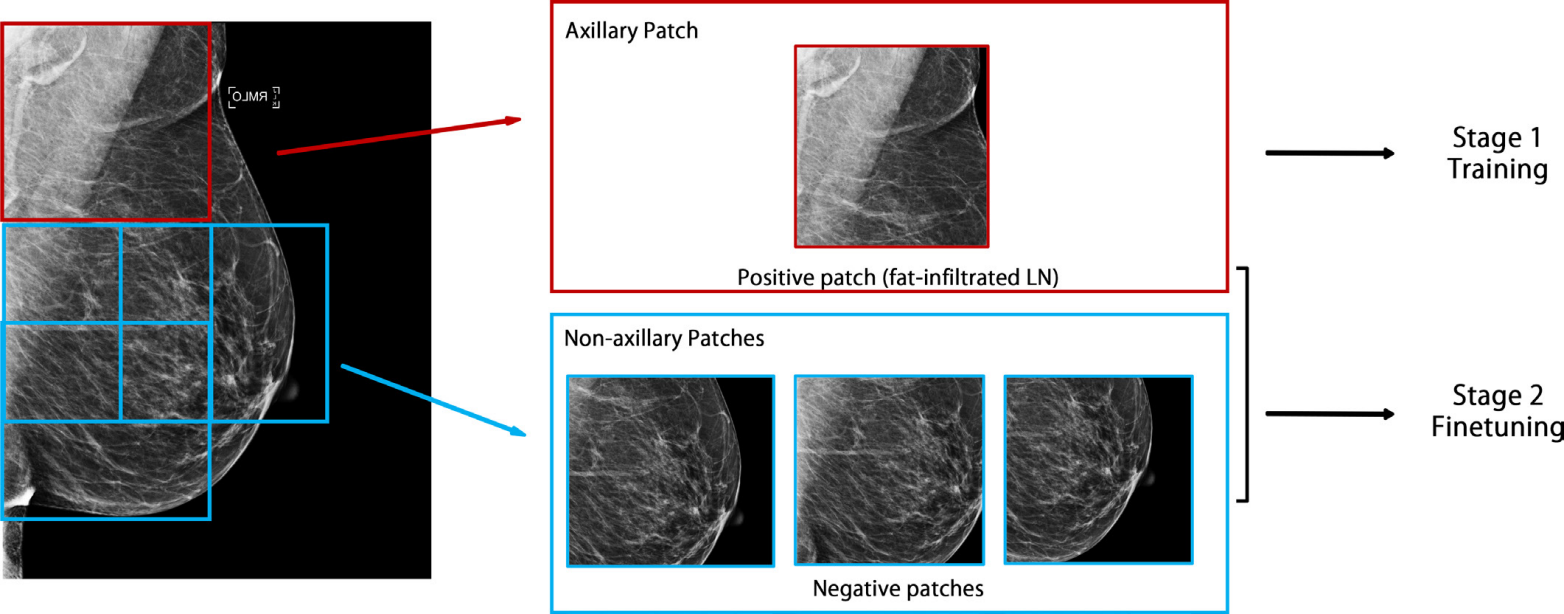
Automated image processing and data set construction. A patch was extracted from the upper outer corner of the MLO view and used as the axillary patch during the model training and hyperparameter tuning stage. Additional patches were extracted by a sliding window approach from the non-axillary region and added to the negative samples in the model-finetuning stage. MLO, mediolateral oblique.

The axillary region of mammograms contains radiographic densities that are unrelated to the imaging characteristics of the axillary LNs. These densities, including but not limited to breast tissue, vessels, muscle, skin folds, fibroglandular tissue and radiographic densities that are also present in the non-axillary portion of the mammogram. To help the model better distinguish the LNs from non-nodal radiographic densities, we sampled patches from non-axillary regions of the breast and used them as additional negative samples. We implemented a sliding-window approach to sample additional 1500 × 1500 pixel images outside the axillary region, with a step size of 750 pixels, as illustrated in Figure 2. These additional non-axillary patches were combined into the negative samples of the initial data set.

Finally, we randomly split the data set into a 70% training set, a 10% development set for hyperparameter tuning, and a 20% testing set. Table 1 shows the class distribution of the initial data set’s training, development and testing sets, and the oversampled larger data set that included the non-axillary patches.

**Table 1.** The distribution of mammograms in the training, development and test sets

	Training	Development	Testing	Total
Fat-infiltrated/positive (axillary)	266	40	110	416
Normal/negative (axillary)	267	49	154	470
Nonaxillary/negative	1291	217	647	2155
Total (axillary only)	533	89	264	886
Total (both axillary and non-axillary)	1824	306	911	3041

### Model training

The model training was conducted in two stages, a model training and selection stage, followed by a model-finetuning stage. In the first stage, various CNN models were trained on the smaller data set with only axillary patches to select the best-performing model architecture and hyperparameter settings. We experimented with several well-established CNN architectures, including VGG16,^21^ ResNet18^22^ and DenseNet121,^23^ with initial weights pre-trained on the ImageNet data set.^24^ Kaiming weight initialization^25^ was used for the binary output fully connected layer. Hyperparameters, including batch size (8 or 16) and initial learning rate ( $1\times 10^{-4}$ , $5\times 10^{-5}$ , or $2.5\times 10^{-5}$ ), were selected by a grid search to optimize the cross-entropy loss. The model with the lowest loss on the development set was selected for the fine-tuning stage. In the second stage, the pre-trained model chosen from the previous stage was further fine-tuned on a larger training data set that included the oversampled negative patches using the best hyperparameters selected from the first stage. In both stages, the model parameters were optimized using the ADAM optimizer^26^ with a step learning rate decay of 0.5 every 15 epochs throughout the training. The training ended when the development set loss did not show improvement for 30 consecutive epochs.

Before training, grayscale image patches were resized to 224 × 224 pixels and converted to 3-channel images by repeating it three times to fit into the pre-trained model that operates on RGB images. Images were standardized to have a of zero and a standard deviation of one. To expand the training data’s size and improve the model’s generalizability, we used data augmentation on the training data, including random 90 degree rotation, random horizontal and vertical flipping, sharpening, noise addition, and contrast variation. The details of data pre-processing and augmentation implementation are described in Supplementary Material 1. The model training process was conducted on NVIDIA Titan Xp graphical processing units (GPUs) with 12 GB of memory and was implemented using the PyTorch framework.^27^

Supplementary Material 1.

### Internal model evaluation and visualization

We evaluated the performance of the final model on the test set by calculating the accuracy, sensitivity, specificity, precision, sensitivity, F1 score and area under the receiver operating characteristic curve (AU-ROC). Confidence intervals were calculated with 10,000 bootstrap resampling. To address the interpretability of our DL model, we generated the visualization of our model outcome on the images using Gradient-weighted Class Activation Mapping (Grad-CAM).^28^ The resulting heatmaps highlighted the most indicative region of the images based on the region’s influence on the model’s prediction outcome.

### External validation

To assess the generalizability of our model, we evaluated it on images from the Curated Breast Imaging Subset of the Digital Database for Screening Mammography (CBIS-DDSM). This data set contains scanned film mammography studies in DICOM format. From the data set, 110 normal mammograms without any abnormalities (Breast Imaging Reporting and Data System (BI-RADS) category 1) and with visible axillary LNs were sampled as the external test set for this study. A breast radiologist, and a graduate student with 4 years of experience in fat-infiltrated LN radiology, independently reviewed the cases and labeled them as fat-infiltrated or normal. Due to the loss of pixel spacing information in this data set, the size of the LN could not be measured directly from the mammograms. Therefore, an LN was labeled fat-infiltrated if it displayed a fat-infiltrated LN morphology with an expanded fatty hilum relative to the surrounding nodal cortex as previously described. Cases with disagreement were further discussed, and a final classification was determined by consensus between the two readers. Cases with low visibility due to multiple overlapping nodes were removed from this external test set. The review yielded 35 cases with fat-infiltrated LNs, and 35 cases with normal axillary nodes, to construct a balanced data set. We evaluated our model on the labeled external data and visualized the model decision with Grad-CAM.

## Results

### Model performance on the internal data set

**Figure 3.**
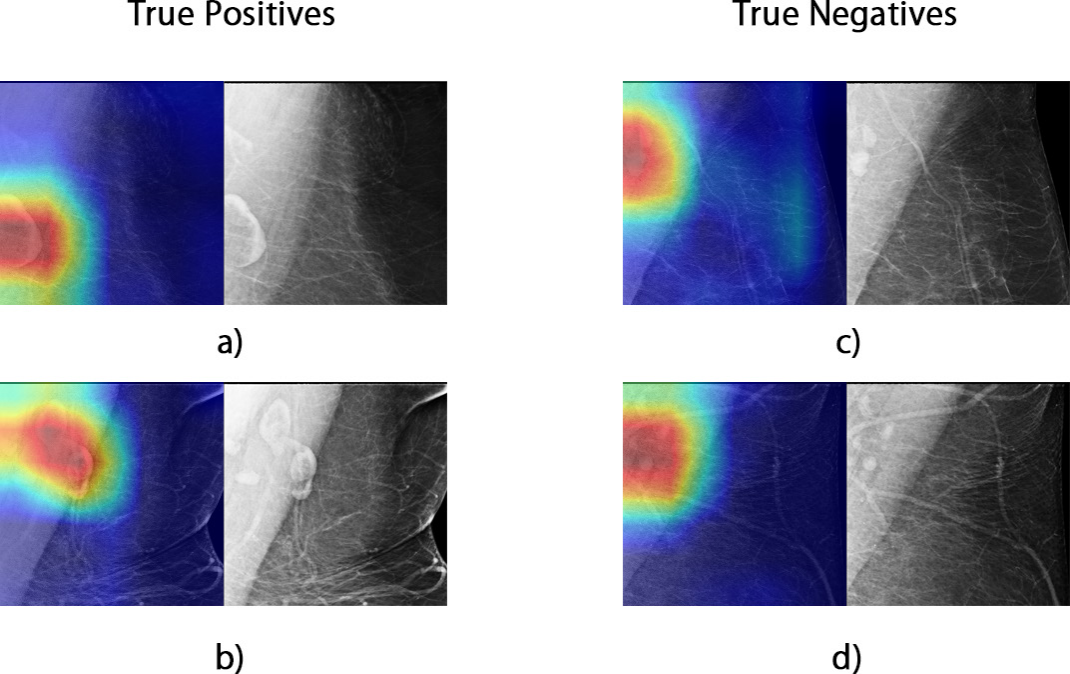
Examples of Grad-CAM visualization. Red highlights the areas that highly influenced the model’s outcome, while blue indicates regions considered unimportant to the predictions by the model. The original grayscale images are shown on the side for reference.

During Stage 1 of training, the ResNet18 model outperformed the other two architectures with a testing accuracy of 0.94 with 15 false predictions out of 264 testing samples (Table 2a). Therefore, the ResNet18 model was chosen for further fine-tuning. The model performance was improved by fine-tuning the model on the oversampled negative patches from the nonaxillary region with a testing accuracy of 0.97 on the axillary patches. The final hyperparameters for each model, based on our parameter tuning on the development set, are shown in Supplementary Table 1, and the comparison of the Stage 1 model performance is included in Supplementary Figure 1. The visualization of the model’s outcome highlighted by the Grad-CAM heatmap (Figure 3) indicated that the model considered the contributing features of both fat-infiltrated and normal LNs in its decision-making. In addition, the model correctly classified the cases as fat-infiltrated, when both the fat-infiltrated and normal LNs were present on the mammograms, by focusing on only the regions of the fat-infiltrated node (Figure 3b).

**Table 2.** Summary of the evaluation metrics of best-performed models on the internal test set with 95% confidence intervals

Model	Accuracy	Precision	Sensitivity	Specificity	F1 Score	AUROC
a) Stage 1 Model performance on the internal test set containing only axillary patches						
Stage 1						
Resnet18	0.94 (0.91, 0.97)	0.94 (0.88, 0.99)	0.93 (0.89, 0.98)	0.96 (0.92, 0.99)	0.93 (0.90, 0.97)	0.98 (0.97–1.00)
VGG16	0.93 (0.89, 0.96)	0.90 (0.84, 0.95)	0.92 (0.87, 0.97)	0.93 (0.89, 0.97)	0.91 (0.87, 0.95)	0.98 (0.97–0.99)
Densenet121	0.92 (0.88, 0.95)	0.89 (0.84, 0.95)	0.91 (0.85, 0.96)	0.92 (0.88, 0.96)	0.90 (0.86, 0.94)	0.97 (0.94–0.99)
Stage 2						
Resnet18	0.97 (0.94, 0.99)	0.95 (0.90, 0.98)	0.97 (0.93, 1.00)	0.96 (0.93, 0.99)	0.96 (0.93, 0.98)	1.00 (0.99–1.00)
b) Model performance on the internal test set containing both axillary and non-axillary patches						
Stage 1						
Resnet18	0.85 (0.81–0.89)	0.45 (0.33–0.57)	0.95 (0.86–1.00)	0.84 (0.79–0.89)	0.61 (0.49–0.72)	0.97 (0.91–1.00)
Stage 2						
Resnet18	0.99 (0.98–1.00)	0.95 (0.86–1.00)	0.97 (0.90–1.00)	0.99 (0.98–1.00)	0.96 (0.92–1.00)	1.00 (1.00–1.00)

AUROC, area under the receiver operating characteristic.

(a) Stage 1 Model performance on the internal test set containing only axillary patches. (b) Model performance on the internal test set containing both axillary and non-axillary patches.

**Figure 4.**
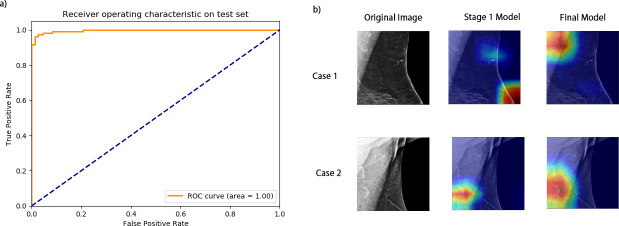
Stage 2 model AU-ROC and examples of improved model predictions. (**a**) The AU-ROC of the Stage 2 model improved to 1.00. (**b**) Two cases illustrate the improved final model performance. In Case 1, the final model correctly localized the axillary region, which the Stage 1 model failed to. In Case 2, the final model more accurately highlighted the entire fat-infiltrated LN, while the Stage 1 model only highlighted part of the node. AUROC, area under the receiver operating characteristic

Of note, although the best-performing model from Stage 1 of training achieved a satisfactory classification performance on the axillary patches, the model was prone to make false predictions by misclassifying features unrelated to fat-infiltrated LNs. Through visual inspection and analysis of the model errors, we found that false-positives were caused by misinterpretation of an adjacent dense vessel as an LN cortex or mischaracterization of small normal LNs grouped around central subcutaneous fat as a single larger node with expanded hilar fat. The false-negative classifications were due to the model failing to localize the LN due to dense muscle or breast tissue, or when the model focused only on the normal LNs when a mixture of normal and fat-infiltrated LNs was present. An example visualization of the errors is shown in Supplementary Figure 2. The errors from the Stage 1 Resnet18 model were more prominent when applying the model to the oversampled test set (Table 2b). The model yielded more false-positive classifications by mistakenly classifying breast tissue and vessels as fat-infiltrated LNs. After fine-tuning the model with oversampled negative patches, the errors were significantly reduced (Table 2b), and the AU-ROC was improved to 1.00 (Figure 4).

### Model performance on external validation

**Figure 5.**
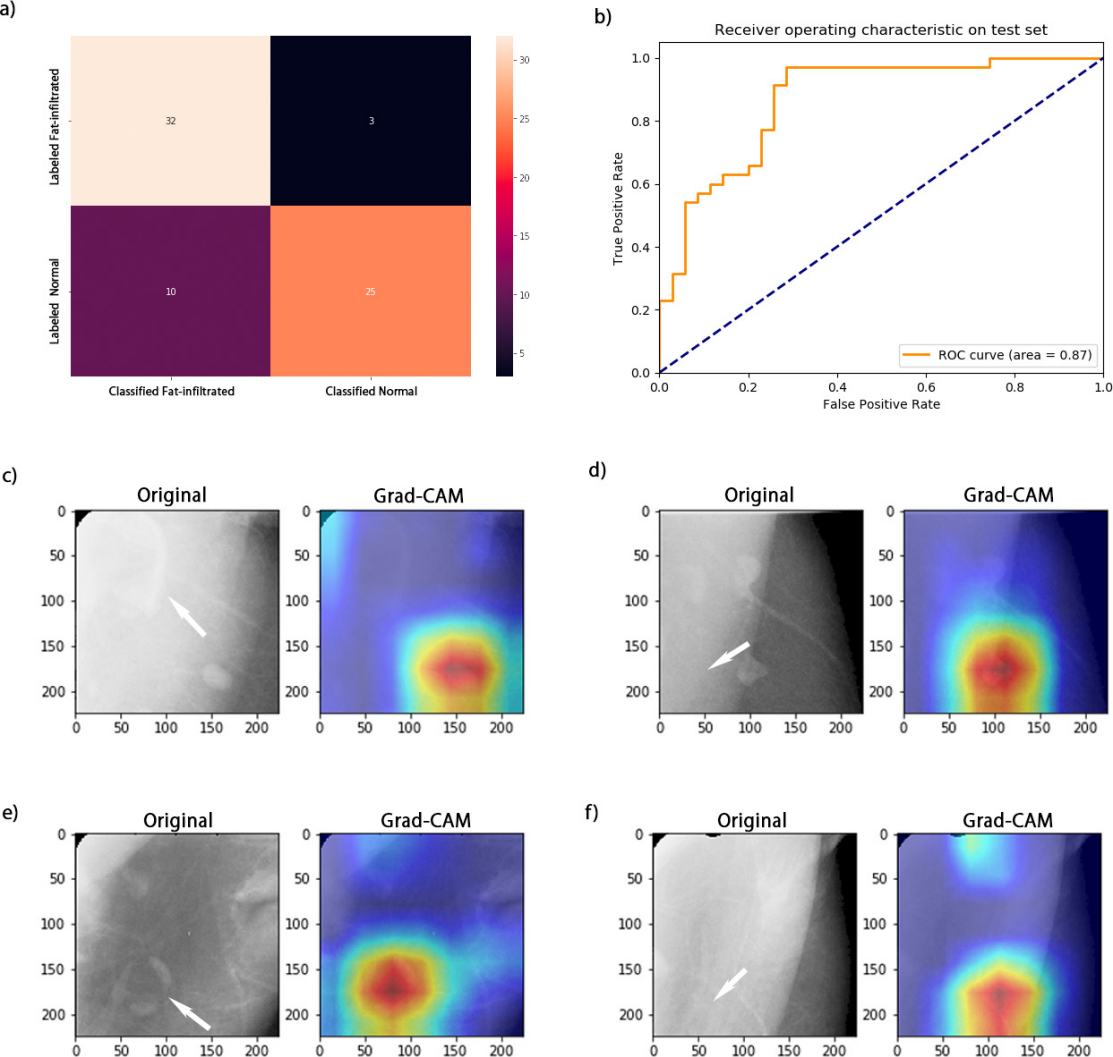
Visualization of model performance and error analysis on the external DDSM data set. (**a**) Confusion matrix of model classification. (**b**) ROC curve of model prediction. (**c**) A false-negative classification resulted from misidentifying the fat-infiltrated LN (arrow-pointed) due to a low contrast from the background and focusing only on the normal node. (**d**) A false-positive case possibly related to the overlying pectoralis muscle and a small node with a lucent center (arrow-pointed). The highlighted node has a lucent center with a normal size relative to the surrounding normal node. (**e**) A false-positive case due to misclassification of multiple normal nodes with space in between as fat infiltrated (arrow-pointed). (**f**) Misclassification of vessels (arrow-pointed) as fat-infiltrated nodes. DDSM, Digital Database for Screening Mammography; LN, lymph node; ROC, receiver operating characteristic.

On the external data set, the Pearson correlation between the independent labels of the two reviewers was 0.61 (*p-*value 0.001). Using the consensus labels of the external data, our model achieved an accuracy of 0.82 (95% CI: 0.77–0.86) and an AU-ROC of 0.87 (95% CI: 0.82–0.91) (Table 3), and the visualization of model performance on the external data is shown in Figure 5. Overall, the model identified most of the cases with fat-infiltrated nodes (sensitivity: 0.92, 95% CI: 0.87–0.96). False negatives resulted from failing to identify the fat-infiltrated LN from the background muscle due to low contrast (Figure 5c). The model resulted in more false-positives on the external data set than on the internal data set. The false-positives were mostly due to misclassifying a cluster of multiple normal nodes or surrounding vessels as fat-infiltrated nodes (Figure 5d–f).

**Table 3.** Model performance on the external data set

Model	Accuracy	Precision	Sensitivity	Specificity	F1 Score	AUROC
Resnet18 (Stage 2)	0.82 (0.77, 0.86)	0.76 (0.69, 0.83)	0.92 (0.87, 0.96)	0.72 (0.64, 0.79)	0.81 (0.78, 0.88)	0.87 (0.82, 0.91)

AUROC, area under the receiver operating characteristic.

## Discussion

Emerging evidence shows that fat-infiltrated axillary LNs are associated with an increased risk of obesity-related pathology.^9,10,13^ Therefore, large-scale studies are needed to confirm axillary fatty nodes as a potential imaging biomarker of obesity-associated diseases. In this study, we developed an automated end-to-end DL-based pipeline, from image pre-processing to the classification of the fat-infiltrated axillary LN status, from digital screening mammography without manual annotation of the LNs. Our model achieved an accuracy of 0.97 and a 1.00 AUROC in classifying the LN status of an independent internal test data set. The model’s accuracy and AU-ROC on the external data set are 0.82 and 0.87, respectively. The model successfully extracted meaningful LN-related features from the mammograms, and was capable of discriminating LN features from other imaging features with similar radiographic density, including breast tissue, pectoralis muscle, skin folds, and vessels. The model benefited from fine-tuning on oversampled training patches from regions containing these look-alike features. Therefore, this automated approach can categorize the status of fat-infiltrated LNs in digital mammograms in bulk with minimal manual intervention and provide a critically needed tool for further research of fat-infiltrated LNs and their association with obesity-related diseases.

Screening mammography is widely used as a primary tool for early breast cancer detection and has a high utilization among age-eligible females in the United States.^29^ Digital imaging studies contain information that is unrelated to the study indication but may be used for patient risk stratification and prediction of future adverse health events. Consideration of these incidental imaging features is referred to as opportunistic imaging. Recent studies have revealed that the added value of opportunistic imaging can improve disease screening and benefit patient care.^30^ For example, studies have reported that breast arterial calcification (BAC) detected by mammography screening could be a risk marker for coronary artery disease.^31–33^ As a result, many computational approaches have been developed to automatically detect BACs from screening mammography.^34,35^ Our automated approach for classifying fat-infiltrated LNs is needed to perform additional studies and determine whether fatty nodes may similarly add extra value to mammography screening as a risk marker for obesity-related disorders.

To date, only a few modest studies have looked at fat-infiltrated LNs visualized on screening mammography. There remains a need for a rapid and generalizable tool to assess the status of fat-infiltrated axillary LNs for future studies. To our knowledge, our study is the first to analyze fat-infiltrated axillary LNs using an automated DL approach. In previous studies, human-extracted features, including expanded hilar fat morphology, hilar and cortical dimensions, and overall LN size, were manually assessed by multiple radiologists.^9,11,14^ The measurement and assessment of fat-infiltrated LNs are not readily available in any existing data set and are resource intensive and time-consuming to collect in bulk. Our end-to-end DL approach has the advantage of automatically learning and extracting radiomic feature representations related to fat-infiltrated LNs directly from mammography images and providing consistent and accurate outcomes on new images. Our model bypasses the manual feature engineering step and requires minimal pre-processing steps, thereby significantly reducing individual bias and the time needed to measure and evaluate the LN status on images. Our model successfully captured the features relevant to the axillary LNs in almost all cases that were verified by Grad-CAM visualizations, and generated the proper classification on our test sets.

The high sensitivity of 0.92 of our model on the subset of the external CBIS-DDSM data set confirmed the model’s utility in classifying fat-infiltrated LNs on mammograms from an unseen data distribution. There are several possible explanations for the reduced overall model performance on the external data compared to the internal data set. DDSM is a collection of digitized analog film-screen mammograms first released in 1997. Due to the inherently low contrast resolution of film screen mammography compared to digital mammography, axillary LNs were more challenging to characterize on the external data set.^36^ Second, the CBIS-DDSM data contain no pixel spacing information and therefore did not allow for an LN measurement as a feature of fat-infiltrated LNs. The labeling of the external data set relied on the LN morphology without consideration of the LN size and was therefore less informative and more error-prone than the internal data set. However, our model performed well in characterizing axillary LNs as fatty on the consensus labels of the external data set using only morphologic criteria. Nonetheless, CBIS-DDSM is currently the only public data set with a sufficient number of digitized full-field mammograms for external validation. Our model should be re-evaluated when additional digital mammography data sets become publicly available.

The results of this study must be considered with a few limitations. First, all mammography exams used for model development were obtained from a single medical center. Our pipeline with a fixed-sized crop of the axilla worked well in capturing the axillary LNs within a uniform image data set; however, due to variations in scanning procedures and equipment, the same setting may not work for mammograms acquired from other institutions. Further training and validation of our model on larger data sets across multiple institutions would improve the generalizability of our approach. Therefore, we plan to further validate our model on additional data from external collaborators. Second, our model coarsely localized LNs based on image-level labels and still generated false-positive classifications in cases where multiple normal LNs overlapped and formed a cluster around subcutaneous fat. The model may benefit from segmentation approaches, such as U-Net,^37^ to identify individual LNs if detailed pixel-level annotations become available. Last, our model was designed to classify the binary status of LNs as normal *vs* fat-infiltrated, and the size cut-off between the two classes was selected based on the evidence and previous findings in this domain.^9^ In future work, we plan to expand the model to provide a more fine-grained classification of fat-infiltrated LNs, including the degree of hilar fat expansion and the number of fat-expanded LNs. We also plan to extend our study to examine the potential associations between fat-infiltrated LN-related features and breast cancer outcomes, and evaluate the model’s viability as a potential risk stratification tool for obesity and other obesity-related diseases. In summary, our study demonstrated a promising performance of a DL model developed to classify mammographically visualized axillary LNs. The model achieved a high performance on both internal and external test sets. This approach can enable further investigation of the role of fat-infiltrated axillary nodes in obesity-associated deaths.
